# Histograms of Oriented 3D Gradients for Fully Automated Fetal Brain Localization and Robust Motion Correction in 3 T Magnetic Resonance Images

**DOI:** 10.1155/2017/3956363

**Published:** 2017-01-30

**Authors:** Ahmed Serag, Gillian Macnaught, Fiona C. Denison, Rebecca M. Reynolds, Scott I. Semple, James P. Boardman

**Affiliations:** ^1^MRC Centre for Reproductive Health, University of Edinburgh, Edinburgh, UK; ^2^Clinical Research Imaging Centre, University of Edinburgh, Edinburgh, UK

## Abstract

Fetal brain magnetic resonance imaging (MRI) is a rapidly emerging diagnostic imaging tool. However, automated fetal brain localization is one of the biggest obstacles in expediting and fully automating large-scale fetal MRI processing. We propose a method for automatic localization of fetal brain in 3 T MRI when the images are acquired as a stack of 2D slices that are misaligned due to fetal motion. First, the Histogram of Oriented Gradients (HOG) feature descriptor is extended from 2D to 3D images. Then, a sliding window is used to assign a score to all possible windows in an image, depending on the likelihood of it containing a brain, and the window with the highest score is selected. In our evaluation experiments using a leave-one-out cross-validation strategy, we achieved 96% of complete brain localization using a database of 104 MRI scans at gestational ages between 34 and 38 weeks. We carried out comparisons against template matching and random forest based regression methods and the proposed method showed superior performance. We also showed the application of the proposed method in the optimization of fetal motion correction and how it is essential for the reconstruction process. The method is robust and does not rely on any prior knowledge of fetal brain development.

## 1. Introduction

**Figure 1:**
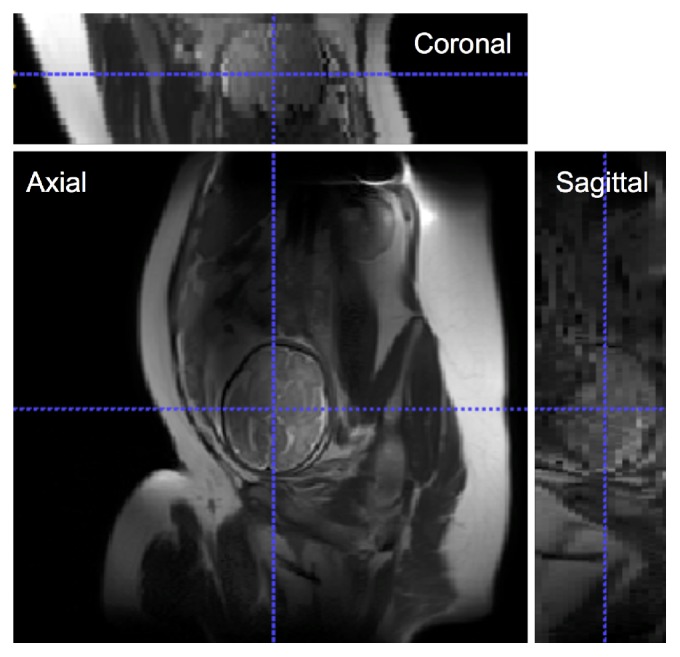
An example of a fetal MR scan at 35.5 weeks of gestational age (GA). The scan shows that the acquired image has an arbitrary fetal orientation, motion artifacts, and low spatial resolution.

Recent successful application of magnetic resonance imaging (MRI) has provided us with an unprecedented opportunity to study, in intricate detail, the developing brain in the living fetus or neonate [1–7]. However, quantitative fetal brain analysis remains challenging due to the unique confrontations associated with fetal MRI such as motion artifacts, low signal, and spatial resolution as shown in Figure 1.

Advances in medical image processing techniques have facilitated the reconstruction of motion-corrected high-resolution 3D fetal MR images [8–12] from stacks of 2D intersecting images, which in turn have laid the foundation for modeling [13–15] and quantitative analysis [1, 15, 16] of the developing fetal brain. Such reconstruction methods rely on initial localization and cropping of the brain region from a standard wide field of view (FOV) MRI, to assist the slice-to-volume registration process [17] by excluding surrounding maternal tissues that can result in registration failure.

Fetal brain localization as an automated processing step is one of the biggest obstacles in expediting and fully automating large-scale fetal MRI processing and analysis. To date, the approaches proposed to address the issue of automating fetal MRI brain localization can be classified into two categories: template matching and machine learning approaches. In the earlier category, Anquez et al. [18] proposed an approach that starts by detecting the eyes using 3D template matching, followed by segmentation of the brain using a 2D graph-cut segmentation of the mid-sagittal slice rotated at several angles. The best matching segmentation is selected and used to initialize a 3D graph-cut segmentation. Taimouri et al. [19] proposed another template matching approach that registers a template to each slice of the fetal brain in 3D.

Examples of machine learning approaches include the method proposed by Ison et al. [20], which is based on a two-phase random forest (RF) classifier to suppress the influence of maternal tissues and provide likely positions of tissue centroids inside the brain. An approximation of a high-order Markov Random Field (MRF) finds the optimal selection of landmarks, and these landmarks, along with a confidence weighted probability map, provide an estimate of the center of a region of interest around the brain. Keraudren et al. [21] described a different learning-based approach that decomposes the search space by performing a 2D detection process, based on Scale-Invariant-Feature-Transform (SIFT) features, before accumulating the votes in 3D. The approach relies on providing the gestational age as a prior knowledge to remove the scale component.

Recently, Kainz et al. [22] proposed a localization method where rotation invariant feature descriptors (based on Spherical Harmonics) for medical volumes of the uterus are calculated in order to learn the appearance of the fetal brain in the feature space. Alansary et al. [23] proposed a different localization method where superpixels are first extracted and a histogram of features for each superpixel is computed using bag of words based on dense SIFT descriptors. Then, a graph of superpixels is constructed and a RF classifier is trained to distinguish between brain and nonbrain superpixels.

Although most previously reported methods achieved accurate brain localization results, all methods were implemented and tested using 1.5 T MR images and the generalization of these approaches to 3 T MR images has not been examined. Fetal MRI performed at 3 T is emerging as a promising modality for the evaluation of fetal anatomy as it provides an increased signal-to-noise ratio (compared with 1.5 T). This increase in the signal-to-noise ratio can allow for decreased acquisition time, increased spatial resolution, or a combination of both, with the overall goal of obtaining more detailed imaging of fetal anatomy.

In this article, we focus on developing a novel method for fully automatic fetal brain localization from raw 3 T MR images, with no need for preprocessing (such as intensity inhomogeneity correction) or prior knowledge about the dataset under study (such as gestational age (GA) or imaging plane). We treat the problem as a machine learning problem where a model is trained using a sample of positive and negative examples. From each sample, discriminant features are extracted, for both positive (brain) and negative (nonbrain) examples, based on a 3D extension of the successful Histogram of Oriented Gradients (HOG) [24] feature descriptor. A sliding window classifier, based on Support Vector Regression (SVR), is used to assign a score to all possible windows in an image and the window with the highest score is selected.

The remainder of this article is organized as follows.  Section 2 describes in detail the proposed method. In Section 3, the experimental results of applying the proposed method to 3 T MR fetal images are presented. We also compare the performance of our method against a template matching method and present its application in the optimization of fetal motion correction.  Section 4 discusses our study and outlines areas for prospective work. This paper is an extension of previous preliminary work [25].

## 2. Methods

### 2.1. Data

The database for this study included a total of 104 MR images from 32 singleton fetuses of healthy women and women with diabetes, between 34.30 and 37.60 weeks of gestational age (mean and standard deviation of 35.92 ± 0.83). Ethical approval was obtained from the National Research Ethics Committee (South East Scotland Research Ethics Committee) and written informed consent was obtained.

### 2.2. MR Image Acquisition

Images were acquired on a Siemens Magneton Verio 3 T MRI clinical scanner (Siemens Healthcare GmbH, Erlangen, Germany). T2-weighted half-Fourier acquisition single-shot turbo spin-echo images were acquired of the fetal brain in sagittal, coronal, and transverse orientations where at least one stack is available in each anatomical direction (HASTE: TR/TE = 1800/86 ms, FOV = 400 × 400 mm, matrix = 192 (phase) × 256 (frequency), and voxel size = 1.5 × 1.5 × 6 mm).

### 2.3. The HOG Descriptor for 2D Images

In the 2D image domain, successful methods such as Harris-Corner detector [26] and the well-known SIFT descriptor [27] rely on aggregated gradients. However, the HOG descriptor [24] organizes gradients into histograms. As the first step, the gradient image is computed by convolving the input image with an appropriate filter mask. A grid of histograms is then constructed, where each histogram organizes the respective gradients into bins according to their orientation. To preserve locality, a histogram is computed for each cell in an evenly spaced grid. Accordingly, each cell contains the same number of gradients (depending on the cell size) and gets assigned exactly one histogram. The cells themselves are then organized in rectangular blocks, which may overlap. The histogram values of all cells within one block are concatenated to form a vector. The vector of each block is then normalized and subsequently, the concatenation of all those block-vectors yields the final feature vector. Further details and an evaluation of the 2D HOG descriptor can be found in Dalal and Triggs [24].

### 2.4. Extending HOG to 3D Images

Due to the nature of the analyzed data, that is, 3D volumetric images, 3D feature descriptors are ideal. It also has been demonstrated that 3D descriptors are much more descriptive than their corresponding 2D, as richer information is encoded into the histograms [28].

Here, our extension of the HOG approach to 3D medical images consists of two steps. First, we need to extract gradients from the images. Second, we need to organize these three-dimensional gradients into bins using appropriate histograms computed over uniformly spaced grid-blocks. This step is straightforward, as we simply extend the grid and histogram dimension, each by one. We then can convert each gradient into spherical coordinates (1) and bin it according to its orientation (azimuth *θ* ∈ [0,2*π*) and zenith *ϕ* ∈ [0, *π*]).

(1) $$\begin{array}{c} \left(\begin{array}{c} r\\ \theta \\ \phi \end{array}\right)=\left(\begin{array}{c} \sqrt{x^{2}+y^{2}+z^{2}}\\ \tan^{-1}\left(\frac{y}{x}\right)\\ \cos^{-1}\left(\frac{z}{r}\right) \end{array}\right). \end{array}$$

The first step, however, does not generalize as easily. To compute the image-gradient, several approaches might be considered. According to Dalal and Triggs [24], the convolution of the image with a 1D [−1, 0, 1] filter mask is most suitable. This approximates the partial first-order derivative according to

(2) ∇fx,y=δfδx,δfδyT

(3) ≈fx+1,y−fx−1,yfx,y+1−fx,y−1,

(4) ∇fx,y,z=δfδx,δfδy,δfδzT

(5) ≈fx+1,y,z−fx−1,y,zfx,y+1,z−fx,y−1,zfx,y,z+1−fx,y,z−1.

Based on the description above, Algorithm 1 summarizes the extraction of the 3DHOG feature vector from 3D fetal MR images.

It is worth mentioning that Histograms of Oriented 3D Gradients have been previously reported in the literature, for example, in the areas of video sequence analysis [29, 30] or 3D object retrieval [31]. Those 3D extended HOGs are different from ours as their data representation was 2D + time [29, 30] or 3D mesh models [31]. Therefore, the previously proposed extensions of HOG cannot be directly (or simply) applied to our data as we deal with 3D volumetric medical images.

### 2.5. Brain Localization Using Sliding Window

A sliding window is used to move over all possible windows in an image, and the window with the highest score is selected. Given that one of our aims is to provide a method that does not require any prior knowledge, we use a sliding window of a fixed size that is large enough to hold the largest brain in our database. To assign a score to each window, we use Support Vector Regression (SVR). For our experiments, the detector has the following default settings: [−1, 0, 1] gradient filter with no smoothing; 9 orientation bins in 0–180 degrees for azimuth and zenith; 3 × 3 × 3 voxel blocks with an overlap of half the block size;* L*2-norm block normalization; 40 × 40 × 5 step size for sliding window; SVR (*C* = 1, *ϵ* = 0.1) with a first-order polynomial kernel. In the Results, we perform an evaluation to measure the performance with respect to the variation of these parameters and to find the best values for our learning task.

### 2.6. Comparisons against Other Methods

We implemented a basic method of template matching, with the template representing an average fetal brain, to compare our proposed method against. We will call the template *T*(*x*_*t*_, *y*_*t*_, *z*_*t*_), where (*x*_*t*_, *y*_*t*_, *z*_*t*_) represent the coordinates of each voxel in the template. We will call the input or search image *S*(*x*, *y*, *z*), where (*x*, *y*, *z*) represent the coordinates of each voxel in the search image. We then simply move the center (or the origin) of the template *T*(*x*_*t*_, *y*_*t*_, *z*_*t*_) over each (*x*, *y*, *z*) point in the search image and calculate the sum of products between the coefficients in *S*(*x*, *y*, *z*) and *T*(*x*_*t*_, *y*_*t*_, *z*_*t*_) over the whole area spanned by the template. As all possible positions of the template with respect to the search image are considered, the position with the best score is the best position. Here, the best score is defined as the lowest Sum of Absolute Differences (SAD):

(6) SADx,y,z=∑i=0N ∑j=0M ∑k=0LDiffx+i,y+j,z+k,i,j,k,

where *N*, *M*, and *L* denote the sizes of each dimension of the template image. We also experimented replacing the support vector regressor with a random forest regressor [32] using the following parameter settings: number of trees in the forest = 10, minimum number of samples required to be at a leaf node = 1, and unlimited number of leaf nodes.

### 2.7. Validation

Leave-one-out and 10-fold cross-validation procedure were performed for the 104 MR images. In case of leave-one-out cross-validation, each image in turn was left out as a test sample and the remaining 103 images were used as the training dataset. In 10-fold cross-validation, the original dataset was randomly partitioned into 10 equal sized subsamples. Of the 10 subsamples, a single subsample was retained as the test data, and the remaining 9 subsamples were used as training data. The cross-validation process was then repeated 10 times (the folds), with each of the 10 subsamples used exactly once as the validation data. A correct detection or complete brain localization was defined as the enclosure of all brain voxels within the detected box.

We also measured the distance *d* between the center of the ground truth bounding box and the center of the detected bounding box, and median absolute error, MdAE, was obtained using the following formula:

(7) $$\begin{array}{c} \text{MdAE}=\underset{i=1,\ldots ,n}{median}\left|\;d_{i}\;\right|, \end{array}$$

where *n* is the total number of images used for validation.

## 3. Results

Using leave-one-out cross-validation, the proposed method provided 96% success rate in brain localization and MdAE of 6.95 mm. When the 10-fold cross-validation was performed, the method provided comparable results with success rate of 95% and MdAE of 7.73 mm.

### 3.1. Influence of Parameters

**Figure 2:**
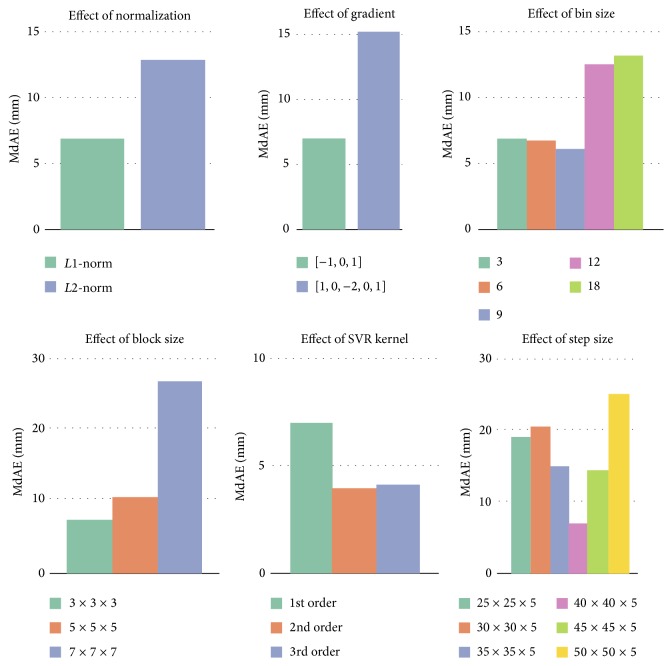
The effect of the various HOG parameters on overall localization performance.

Figure 2 summarizes the influence of the various HOG parameters on overall detection performance. Detector performance is sensitive to the way in which gradients are computed, but the simplest scheme provided better accuracy. As shown in Figure 2, increasing the number of orientation bins did not greatly improve performance up to 9 bins, but beyond this, performance started to degrade. Gradient strengths vary over a wide range due to intensity inhomogeneity; therefore effective local contrast normalization between overlapping blocks is essential for good performance. From our experiments, it turns out that* L*1-norm is better than* L*2-norm. When tested for different block sizes, the block size of 3 × 3 × 3 voxels yielded the best performance. Testing for different sliding window step sizes, the step size of 40 × 40 × 5 had the best performance. Experiments also showed that SVR with a second- and third-order polynomial kernels outperformed linear SVR.

### 3.2. Comparisons against Other Methods

To date, no method has been proposed for automatic localization of the fetal brain using 3 T MR images, making it difficult to compare our proposed method against a benchmark method. However, we compared our results against a basic method of template matching, with the template representing an average fetal brain. The median localization error for the implemented template-based matching approach was MdAE = 12.5 mm. This shows a degradation of brain localization accuracy compared to proposed method using Histograms of Oriented 3D Gradients. When we experimented replacing the support vector regressor with a random forest regressor, we obtained results with MdAE of 7.9 mm.

### 3.3. Application to Robust Motion Correction

**Figure 3:**
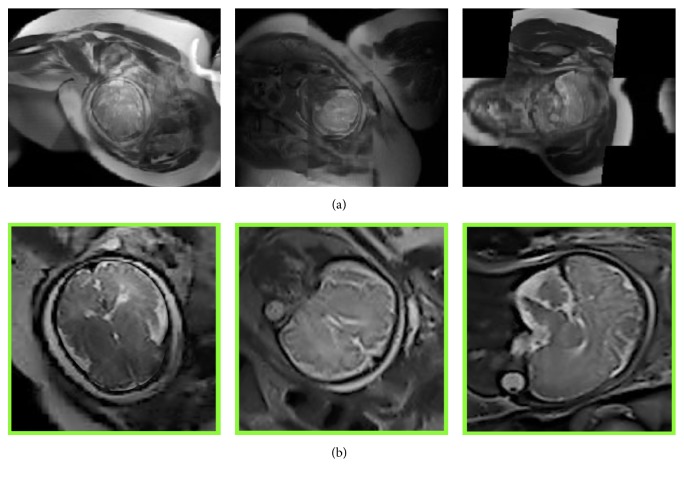
Three MRI scans reconstructed using raw MRI without brain localization and cropping (a). Inside the green colored frames (b), the same images were motion-corrected after automatic brain localization and cropping using the proposed method.

Due to the nature of the acquisition, that is, acquiring stacks of 2D slices in real-time MRI, in order to reduce the scan time while avoiding slice cross-talk artifacts, slices are quite often misaligned due to fetal motion and form an inconsistent 3D volume (see Figure 1). Registration-based approaches for reconstructing motion-corrected high-resolution fetal brain volumes require a cropped box around the brain to exclude irrelevant tissues, otherwise the registration, and the reconstruction, will likely fail.  Figure 3 shows an example of three different subjects that were successfully reconstructed using the Baby ToolKit [17]. It is clear that brain cropping is essential for the reconstruction process, and the registration fails when the whole FOV is used.

## 4. Discussion

**Figure 4:**
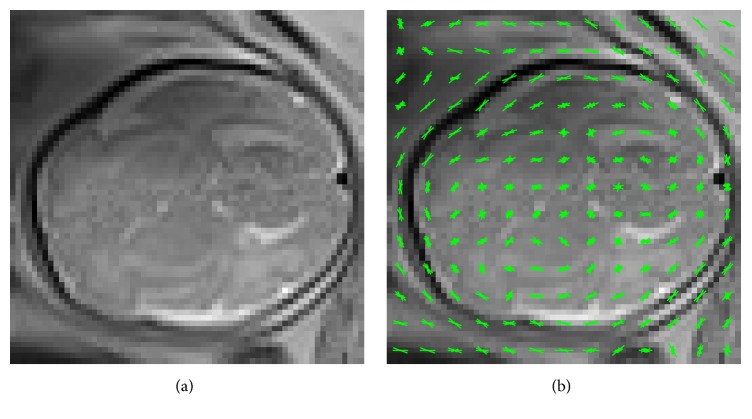
An example of an axial fetal MR image used from the training database (a) and the resulting computed HOG descriptor overlaid on the example image (b).

In this article, Histogram of Oriented Gradients (HOG) is proposed to automatically localize the fetal brain in 3 T MR images. The main contribution of the article is the extension of HOG to 3D for fetal brain MRI localization. We chose to use HOG features partly because of its demonstrated superiority to other widely used features like SIFT/SURF in many applications [24, 33, 34] and partly because the use of HOG features in our task is rational as head boundaries are HOG rich relative to surrounding maternal tissues (see Figure 4).

We also chose to use Support Vector Regression (SVR) on HOG features, instead of Support Vector Machine (SVM), which was proposed by Dalal and Triggs [24] in the original HOG feature descriptor. Typically, SVR is used with a sliding window approach to assign a score to all possible windows in an image and the window with the highest score is then selected. Therefore, by using SVR over SVM, we avoid an inevitable problem that arises in overlapping sliding window approach, which is the occurrence of multiple detection windows in the same neighborhood area [35].

We examined the influence of different HOG parameters on overall detection performance in terms of median absolute error of a bounding box detected around the fetal brain and the ground truth (defined manually). In our results, we achieved 96% for complete brain localization, and the automated detection process takes less than 5 seconds on a normal computer. Our method is more general than Anquez et al. [18] as it does not rely on localizing the eyes. It also does not require any prior knowledge (such as gestational age) as in Keraudren et al. [21].

We carried out comparisons against template matching and random forest based regression methods and the proposed method showed superior performance. We also showed the application of the proposed method in the optimization of fetal motion correction and how it is essential for the motion correction process.

Noteworthy is the fact that the original HOG feature descriptor is not rotation invariant, given that we relied on the large variation of the training dataset to handle different orientations. Using a database of 104 MRI scans aged between 34 and 38 weeks of GA, the rotation variance is represented within the training samples and hence the model will learn to classify it. In addition, the training samples were acquired in different planes (axial, coronal, and sagittal), which is another strength of the proposed method.

## 5. Conclusion

The main motivation behind this work was to automate the fetal brain localization step which is one of the obstacles preventing rapid deployment and full automation of large-scale fetal MRI postprocessing. Due to the nature of the acquisition, that is, acquiring stacks of 2D slices in real-time MRI, in order to reduce the scan time while avoiding slice cross-talk artifacts, slices are quite often misaligned due to fetal motion and form an inconsistent 3D volume. Registration-based approaches for reconstructing motion-corrected high-resolution fetal brain volumes require a cropped box around the brain to exclude irrelevant tissues, otherwise the registration, and the reconstruction, will likely fail. Future work will include applying and evaluating the brain localization framework to a larger GA range (i.e., second trimester), and different MRI modalities of the fetal brain, such as T1-weighted, Echo Planar Imaging (EPI), and Diffusion Tensor Imaging (DTI). This will allow for more accurate assessment and analysis of the developing fetal brain across several stages of development using different imaging modalities. The method is available to the research community at http://brainsquare.org.

## Figures and Tables

**Algorithm 1:**
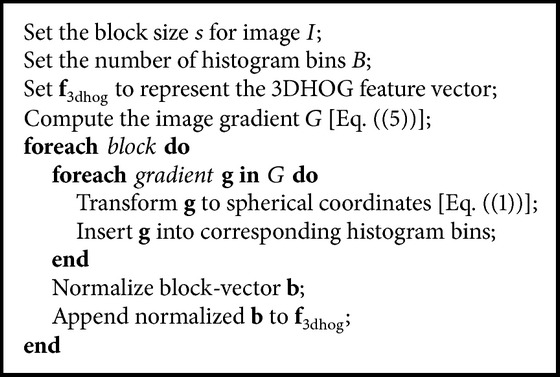
Histograms of Oriented 3D Gradients (3DHOG).
